# Whole breast and excision cavity radiotherapy plan comparison: Conformal radiotherapy with sequential boost versus intensity-modulated radiation therapy with a simultaneously integrated boost

**DOI:** 10.1002/jmrs.4

**Published:** 2013-02-13

**Authors:** Katherine Small, Chris Kelly, Rachael Beldham-Collins, Val Gebski

**Affiliations:** 1 Nepean Cancer Care CentrePenrith, New South Wales 2750, Australia; 2 The Crown Princess Mary Cancer CentreWestmead, New South Wales 2145, Australia; 3 NHMRC Clinical Trials Centre, University of SydneySydney, New South Wales 2050, Australia

**Keywords:** Breast cancer, conformal radiotherapy, intensity-modulated radiotherapy (IMRT), lung dose, radiation therapy

## Abstract

**Introduction:**

A comparative study was conducted comparing the difference between (1) conformal radiotherapy (CRT) to the whole breast with sequential boost excision cavity plans and (2) intensity-modulated radiation therapy (IMRT) to the whole breast with simultaneously integrated boost to the excision cavity. The computed tomography (CT) data sets of 25 breast cancer patients were used and the results analysed to determine if either planning method produced superior plans.

**Methods:**

CT data sets from 25 past breast cancer patients were planned using (1) CRT prescribed to 50 Gy in 25 fractions (Fx) to the whole-breast planning target volume (PTV) and 10 Gy in 5Fx to the excision cavity and (2) IMRT prescribed to 60 Gy in 25Fx, with 60 Gy delivered to the excision cavity PTV and 50 Gy delivered to the whole-breast PTV, treated simultaneously. In total, 50 plans were created, with each plan evaluated by PTV coverage using conformity indices, plan maximum dose, lung dose, and heart maximum dose for patients with left-side lesions.

**Results:**

CRT plans delivered the lowest plan maximum doses in 56% of cases (average CRT = 6314.34 cGy, IMRT = 6371.52 cGy). They also delivered the lowest mean lung dose in 68% of cases (average CRT = 1206.64 cGy, IMRT = 1288.37 cGy) and V20 in 88% of cases (average CRT = 20.03%, IMRT = 21.73%) and V30 doses in 92% of cases (average CRT = 16.82%, IMRT = 17.97%). IMRT created more conformal plans, using both conformity index and conformation number, in every instance, and lower heart maximum doses in 78.6% of cases (average CRT = 5295.26 cGy, IMRT = 5209.87 cGy).

**Conclusion:**

IMRT plans produced superior dose conformity and shorter treatment duration, but a slightly higher planning maximum and increased lung doses. IMRT plans are also faster to treat on a daily basis, with shorter fractionation.

## Introduction

Early-stage breast cancer patients, who receive radiation therapy as an adjunct to breast-conserving surgery, chemotherapy, and hormonal therapy, have improved local control rates comparable with those receiving radical surgery, with established conformal radiotherapy (CRT) of the breast consisting of treatment to the whole-breast tissue using tangential beams prescribed to 45–50.4 Gray (Gy) in 25 fractions.^1^–^4^ A sequential boost to the original tumour site using photons or electrons, prescribed to 10–16 Gy in 5–8 fractions, follows whole-breast irradiation.^5^–^8^

Rather than treating the whole breast and excision cavity boost volumes as separate entities, it is possible to simultaneously integrate the boost volume into the planning process,^9^–^11^ and therefore treat the whole-breast volume and the boost volume concomitantly with CRT. This parallel treatment of both breast volumes results in a shorter fractionation and has not shown to significantly increase acute skin toxicity.^9^,^12^

Intensity-modulated radiation therapy (IMRT) of early-stage breast cancer can increase dose homogeneity while decreasing dose to the normal tissue, compared with conformal sequential breast irradiation.^9^,^13^ Treating the whole-breast volume concurrently with the excision cavity volume as a simultaneously integrated boost (SIB) is also possible using IMRT, and has the added benefit of a shorter fractionation, without causing significant skin toxicity regardless of breast size, resulting in a better cosmetic outcome for the patient,^13^–^15^ and may improve dose to surrounding organs at risk.^16^,^17^

A comparative study was conducted, comparing the difference between a combination of a SIB and IMRT and the traditional conformal two-phase approach, to determine if any technique provides an improvement in plan conformity. It was expected that IMRT SIB plans would deliver more conformal dose around the planning target volumes (PTVs), and give comparative dose to organs at risk, compared with the conformal sequential boost plans.

## Materials and Methods

A comparative study was performed using 25 patient data sets, a conformal breast plan with a sequential boost, and an IMRT SIB plan. The two plans for each patient were then compared to determine if either technique provided a clinical improvement in plan quality. Evaluation criteria consisted of the overall plan maximum dose, maximum heart dose, ipsilateral lung dose, and two conformity indices: (1) Lomax and Scheib healthy tissue conformity index^19^ and (2) van't Riet conformation number.^19^ The results were analysed as a paired *t*-test, and an alpha level set at 5%.

### Patient selection and data acquisition

The data of 25 primary breast cancer (PBC) patients treated at Nepean Cancer Care Centre were used for this study. Fourteen left-sided PBC patients and 11 right-sided PBC patients had been previously treated with breast conservation lumpectomy surgery, and were aged from 38 to 74. The whole-breast volumes ranged from 547.9 to 3319 cm^2^ and tangent separations ranged from 17 to 27.9 cm. The range of breast sizes and shapes is typical of those encountered clinically. Patients excluded from the study were those requiring posterior axilla and supraclavicular fossa treatment and those who did not have excision cavity volumes.

All patients were positioned supine on an in-house inclination board with arms raised above their heads, with head and arms surrounded by a Vacfix immobilization vac bag. A radio-opaque marker was then placed on the lumpectomy scar and skin marking tattoos. Computed tomography (CT) slices were obtained in 2.5-mm slices at 2.5 mm separations on a GE LightSpeed (Schenectady, NY) wide-bore CT scanner. CT data was downloaded and plans were developed using Philips Pinnacle (Amsterdam, the Netherlands) 8.0 m treatment planning system.

### Volume delineation

**Figure 1:**
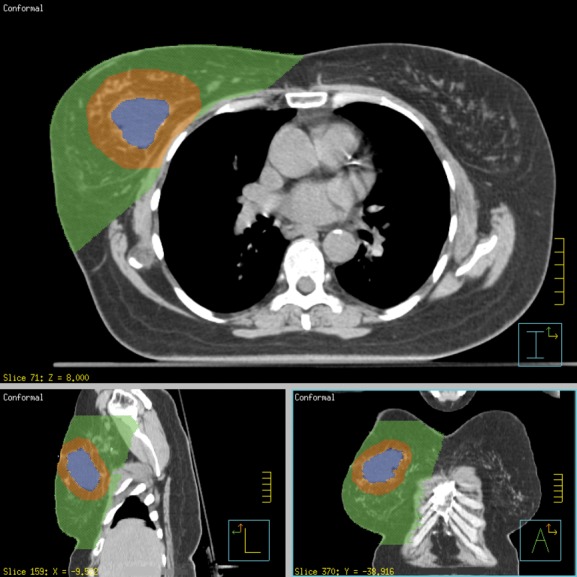
The whole-breast planning target volume (PTV) in green, the excision cavity PTV in orange, with the excision cavity clinical target volume contoured blue.

The treating radiation oncologist contoured the clinical target volume (CTV), the whole-breast PTV, and the excision cavity PTV, covering the breast tissue superiorly by 1.5 cm and beyond the infra-mammary fold by 1.5 cm. The medial border lay at midline, while the lateral border lay at the anterior portion of the latissimus dorsi muscle. The CTV and excision cavity were volumed based on the scar location, surgical reports, mammograms, and the planning CT scan. Figure 1 shows the typical volumes created.

Each lung was contoured from the most cranial to most caudal slice. The heart was also contoured, from the pulmonary trunk to the most caudal slice of the heart and included the encompassing pericardium. A dose grid was placed to include all organs at risk, PTVs, and areas of interest.

### Conformal radiotherapy plans

A mono-isocentric whole-breast technique was used for all plans, with medial and lateral beams selected at angles that created a parallel edge to the posterior portion of the whole-breast PTV. Shielding was created along the posterior portion of the beam, shielding the excess ipsilateral lung and normal tissue using 0.5- and 1-cm multi-leaf collimator (MLC) leaves, with a margin around the posterior portion of the whole-breast PTV of 0.5 cm.

Field sizes were created with a minimum of 2 cm of tissue overshoot anteriorly, a minimally divergent edge along the superior border of the beam by allowing a maximum superior jaw of 2 cm superior to the isocentre. An extra 1 cm of coverage was added to account for the inferior divergence.

Each plan was then manually optimized using 15°, 30°, 45°, or 60° wedges, additional medial or lateral tangents to segment hot spots, direct beams, 18- and 6-MV mixed beams and boost prescriptions to cold areas, and alternate-day 0.5-cm bolus to increase skin dose. Table 1 shows a summary of the different methods used in planning CRT plans.

**Table 1:** Different methods used when planning conformal radiation therapy plans. The total shows the number of patients requiring that specific planning technique

Patient	18-MV beam	0.5-cm alternate-day bolus	Direct beam	Boost prescription	Electrons excision cavity	Photon excision cavity
1						X
2				X		X
3		X				X
4		X		X		X
5						X
6						X
7					X	
8				X	X	
9					X	
10		X				X
11				X	X	
12			X		X	
13						X
14	X					X
15			X			X
16	X		X	X		X
17						X
18						X
19						X
20				X		X
21				X		X
22			X			X
23					X	
24				X		X
25			X		X	
Total	2	3	5	9	7	18

At least 90% of the whole-breast dose was delivered via medial and lateral tangent beams, with a normalization point selected at the centre of the whole-breast PTV as per ICRU^18^ guidelines.

**Figure 2:**
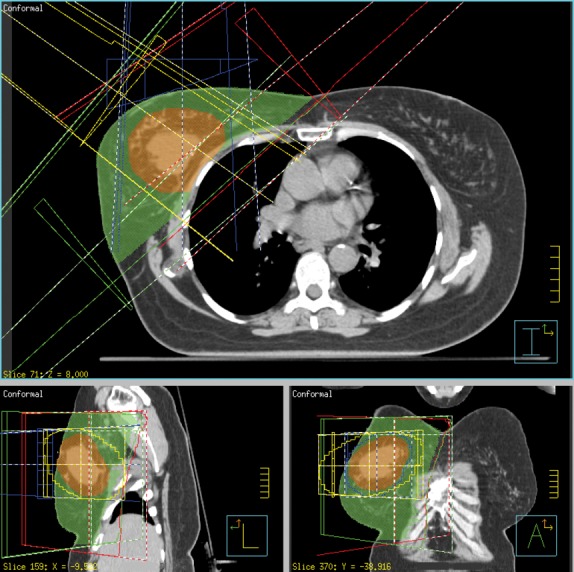
Typical field arrangement for a conformally planned whole breast and sequential excision cavity photon boost.

The excision cavity was then planned sequentially, with a skin appositional electron beam, or a 6-MV photon field arrangement with wedges. The excision cavity boost technique was selected as a best fit for each patient, and it was based on cavity location, depth, and size. Photon field arrangements used 1–3 coplanar 6-MV photon fields, depending on the size and location of the excision cavity PTV, with most using 15°, 30°, 45°, and 60° dynamic wedges. Electron boosts utilized 9, 12, or 15 MeV electron energies in a single skin appositional field. A boost phase normalization point was placed in the centre of the excision cavity volume. Figure 2 shows the typical field arrangement for a whole breast with a sequential single-field photon boost. Adaptive and collapsed cone convolution dose algorithms were used to calculate photon dose, using a full convolution superposition calculation, and three-dimensional models of the planned electron energy were calculated using the Hogstrom algorithm, available in the Pinnacle 3D Planning System.

### IMRT with SIB plans

An IMRT SIB plan was created for each patient data set using medial and lateral beam angles, with field sizes selected similarly to the corresponding conformal plans. All beams were treated with 6-MV photons. A 5- to 6-beam plan was created for each data set. The medial and lateral beams were used as beam weight optimized only open fields. Medial and lateral beams were then created with the same geometry as the tangent beams and assigned to the direct machine parameter optimization (DMPO) dose optimization type. A DMPO-optimized direct field, covering the whole-breast PTV, was also used for one patient with a larger separation.

The DMPO dose optimization type, a feature of the Pinnacle planning system, creates MLC settings during the optimization. Planning time is decreased as conversion; filtering and weight optimization are not necessary with a DMPO optimization.

**Figure 3:**
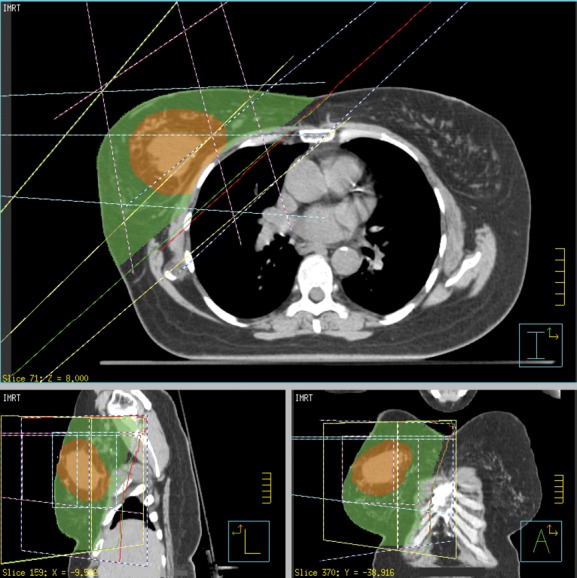
Typical field arrangement for an intensity-modulated radiotherapy planned whole breast and simultaneously integrated excision cavity photon boost.

For the excision cavity PTV, 1–2 photon beams were selected manually and varied according to the size and location of the excision cavity PTV. A field size was then selected to cover the excision cavity PTV with a margin of 0.5 cm while still attached to the same isocentre as the tangent fields, so as to be able to treat the entire volume with one isocentre. Figure 3 displays a typical beam arrangement for an IMRT plan.

Initial weights were selected so as to make the open beam-weight-optimized tangent beams receive 80% of the dose (40% each to the medial and lateral field), and the remainder of the dose to be distributed equally through the optimized tangents and excision cavity PTV beams. After optimization, due to the beam-weight optimization, the total dose delivered through the open medial and lateral tangent beams was approximately 60%, with the remainder distributed through the DMPO-optimized beams.

In order to keep the IMRT SIB plans robust to patient organ motion and setup error, we aim to keep segment size and minimum segment monitor units (MU) fairly high. Plans were performed with minimum segment area of 9 cm^2^ and minimum MU of 6. The first 10 iterations were fluence optimizations, which was then converted into beam segmentations. A maximum of 50 iterations were performed.

The plan was prescribed 60 Gy to excision cavity PTV in 25 fractions, with objectives created at the inverse planning stage to ensure whole-breast PTV received 50 Gy over 25 fractions. The collapsed cone convolution dose algorithm was used to calculate dose, using a full convolution superposition calculation.

### End points

Each plan was evaluated individually, with plans considered acceptable if 95% of 50 Gy was covering the whole-breast PTV, and if 95% of 60 Gy was covering the excision cavity PTV, using two-dimensional and three-dimensional evaluation. Allowances were made for superior and inferior coverage by ensuring the 47.5 Gy isodose line covered the whole-breast PTV at 1.8 cm inferior to the superior (minimally divergent) border with allowances made medially and laterally for the 3–4 cm inferior portion of the whole-breast PTV, due to divergence. Dose to the surrounding tissue was kept as low as practicable. The maximum point dose was kept below 110.5% of the prescribed dose in each PTV.

Measured data for plan comparison included the lung dose to the ipsilateral lung in the form of mean dose, V20, and V30, the proportion of each PTV receiving 95% and 110% of the prescribed doses, the proportion of whole-breast PTV receiving 95% of 60 Gy, and the overall maximum dose. The maximum heart dose was also measured on left-sided PBC patients, as this is a measure commonly used at this clinical institution. Dose to the heart for right-sided PBC patients was not calculated in this study.

The van't Riet^19^ conformation number and Lomax and Scheib^19^ conformity index for the excision cavity were produced. Please refer to Table 2 for index equations. An index value of 1 indicates ideal conformity. The conformity index tends to evaluate coverage but does not take into account any dose to healthy tissue. The conformation number expresses conformity, by taking into account dose to the surrounding healthy tissue, but cannot give any plan information regarding plan heterogeneity.^19^ For this reason, conformity indices are not the only evaluation tools, hence doses to both PTVs, and the surrounding organs at risk were also assessed to create a clearer picture of each plan's overall quality.

**Table 2:** Equations for the conformity indices used in this study. In this study, the reference isodose used was the 57 Gy (95% of 60 Gy) isodose line

Conformity measure	Equation	Legend
Conformity index	TV_RI_/*V*_RI_	TV_RI_, target volume covered by the reference isodose
		*V*_RI_, volume of the reference isodose
Conformation number	(TV_RI_/TV) ×(TV_RI_/*V*_RI_)	TV_RI_, target volume covered by the reference isodose
		TV, target volume
		*V*_RI_, volume of the reference isodose

### Statistical method

All statistics were taken from the Varis (Varian Medical Systems, Palo Alto, CA) patient database and Pinnacle V8.0 m planning system, using dose volume histogram information and volumetric data. The results were analysed using a paired *t*-test using the statistical programme Analysis of Censored and Correlated Data (ACCorD). A resultant *P*-value of <0.05 implied a statistically significant difference between the measures existed.

## Results

Conformal radiotherapy plans delivered the lowest plan maximum doses in 56% of cases (average CRT =6314.34 cGy, range = 6135.7–6501.3 cGy, 95% confidence interval [CI] [6275.133, 6353.467]; average IMRT = 6371.52, range = 6145.8–6616.8 cGy, 95% CI [6329.612, 6413.388], *P* = 0.08) (Table 3).

**Table 3:** The comparable maximum plan doses for conformal radiotherapy (CRT) and intensity-modulated radiation therapy (IMRT) with simultaneously integrated boost plans

Maximum dose parameters	CRT	IMRT
Average maximum dose (cGy)	6314.34	6371.52
Range (cGy)	6135.7–6501.3	6145.8–6616.8
95% Confidence interval	6275.133, 6353.467	6145.8, 6616.8
*P-*value	0.08	0.08

Conformal radiotherapy plans also delivered the lowest mean lung dose in 68% of cases (average CRT =1206.64 cGy, 95% CI [1136.347, 1276.853]; IMRT =1288.37 cGy, 95% CI [1374.581, 2122.219]), V20 in 88% of cases (average CRT = 20.03%, IMRT = 21.73%, 95% CI [18.392, 21.674]), and V30 doses in 92% of cases (average CRT = 16.82%, IMRT = 17.97%, 95% CI [15.146, 18.5036], *P* ≤ 0.001 in all lung dose results) (Table 4).

**Table 4:** The comparable lung doses of conformal radiotherapy (CRT) and intensity-modulated radiation therapy (IMRT) with simultaneously integrated boost plans. All lung dose measurements have a *P-*value ≤0.001

Lung evaluation parameters	CRT	IMRT
Average lung mean dose (cGy)	1206.64	1288.37
V20 (%)	20.03	21.73
V30 (%)	16.82	17.97

Intensity-modulated radiation therapy plans created lower heart maximum doses in 78.6% of cases (average CRT = 5295.26 cGy, range 4858.3–5743.8 cGy, average IMRT = 5209.87 cGy, range 4895.17–5862.8 cGy, 95% CI [5072.616, 5347.184], *P* = 0.08) (Table 5).

**Table 5:** The average maximum heart doses for conformal radiotherapy (CRT) and intensity-modulated radiation therapy (IMRT) with simultaneously integrated boost plans

Heart evaluation parameters	CRT	IMRT
Average maximum heart dose (cGy)	5295.26	5209.87
Range (cGy)	4858.3–5743.8	4895.17–5862.8
*P-*value	0.08	0.08

In all instances, IMRT plans were also considered more conformal when using both conformity index and conformation number. The average CRT plan conformity index was 0.5844 (95% CI 0.557, 0.6107) and conformation number was 0.5665 (95% CI 0.543, 0.589) compared with the average IMRT conformity index 0.7527 (95% CI 0.726, 0.779) and conformation number 0.7386 (95% CI 0.7127, 0.765), *P* ≤ 0.001 in both instances (Table 6).

**Table 6:** The average conformity indices, confidence intervals (CI), and *P-*values for the conformal and intensity-modulated radiation therapy (IMRT) plans

Plan evaluation indices	Conformal radiotherapy	IMRT
Average conformity index	0.5844	0.7527
95% CI	0.557, 0.6107	0.726, 0.779
*P-*value	≤0.001	≤0.001
Average conformation number	0.5665	0.7386
95% CI	0.543, 0.589	0.7127, 0.765
*P-*value	≤0.001	≤0.001

**Figure 4:**
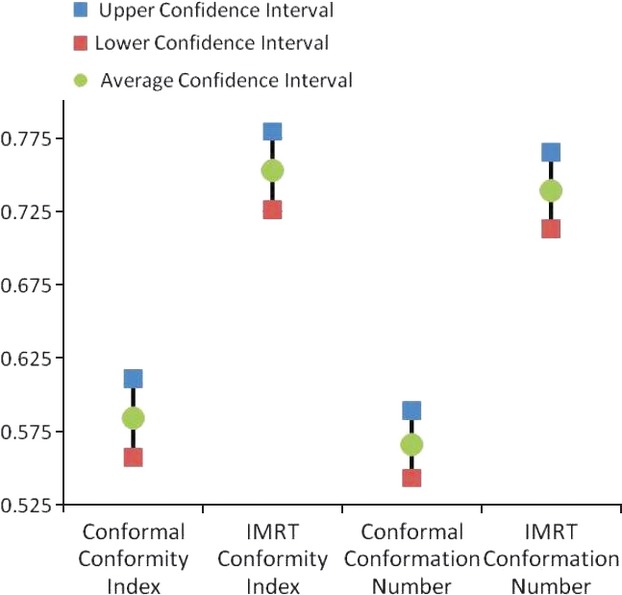
Comparison of conformal radiotherapy and intensity-modulated radiation therapy plan conformity indices for the excision cavity planning target volume.

Figure 4 displays the two conformity indices measured in this study. The green circle displays the average index for the CRT and IMRT plans, while the error bars show the upper and lower 95% CI. The graph indicates a statistically significant difference in plan conformity for the excision cavity PTV.

## Discussion

Breast radiation therapy has always been challenging in terms of the range of sizes and shapes of breast volumes as well as the proximity of the whole-breast volume to the surrounding critical organs. Another inherent challenge when planning whole-breast radiation therapy is the reality that treatment is somewhat limited to what is essentially a parallel-opposed technique in order to avoid these surrounding critical organs.

The IMRT SIB planning technique delivered better plans in terms of dose homogeneity, but delivered poorer quality plans in terms of lung dose. The increased homogeneity, while being considered advantageous, is relatively modest, and does not necessarily indicate an increase in treatment efficacy. The increased lung dose is inconsistent with previous findings, which showed a decrease in lung dose for SIB and IMRT plans.^10^,^20^

It has been suggested that the potential damage caused by an increase in lung dose could possibly outweigh the benefits of surviving breast cancer,^21^ so even modest increases in lung doses should be carefully considered.

For CRT plans, excision cavity depth, size, and location influenced the choice of photons or electrons; however, for IMRT boost fields, 6-MV photons were used exclusively to treat the excision cavity. The increase in lung dose could possibly be attributed to this fact; however, this theory requires further investigation.

While the IMRT SIB plans used only 6-MV photon fields, the CRT plans required more variables to create acceptable plans, like 18-MV beams, electron excision cavity beams, boost doses, and alternate-day bolus to create clinically acceptable plans. The ability to create a comparable plan using only 6-MV photons, rather than the aforementioned variables, can be advantageous to departments in terms of decreased daily treatment time, and when resources such as electrons and higher energies like 18 MV are not available on every linear accelerator. The ability to create plans while only using 6 MV may also mean that planning time is reduced as there are fewer variables to consider when creating a plan.

Intensity-modulated radiotherapy SIB plans require less treatment time each day, as there is no need to enter the treatment room to add or change accessories. The overall treatment duration is also reduced, as the boost dose is delivered concomitantly thereby reducing treatment in these instances by 5 treatment days. This is beneficial to the patient, as they have to attend treatment for 5 fewer days, and to the radiation therapy department, as patient throughput is increased.

No extra work was required from the radiation oncologist to create IMRT plans, as all IMRT contours were based around the standard breast and breast boost contours; however, the IMRT plans require additional quality assurance testing by physics.

The IMRT technique can be extended to other breast treatment scenarios, such as whole-breast treatment without a boost volume, which could benefit the patient by reducing daily treatment time, and increasing plan homogeneity. The IMRT SIB technique can also be utilized on bilateral breast volumes with and without boost volumes, treated with a single isocentre.

While the IMRT SIB plans delivered a lower maximum heart dose in 78.6% of patients, a *P-*value of 0.08 means that this finding is not statistically significant, and is in keeping with several studies.^22^–^24^ The mean heart dose was not measured in this study, but could be more informative than a maximum dose, as it takes into account the total heart volume rather than one point within the heart. A literature review by Abeyaratne^25^ showed that breast IMRT plans could reduce high doses to the heart, but increased the amount of middle to low dose delivered to the heart. Efforts such as inspiratory breath hold, currently used in our department, may further reduce cardiac dose in treatment of the left breast.^26^ The potential to reduce radiation dose to the heart is an important consideration, as some studies using radiobiological models found a reduced risk of the probability of cardiac tissue complications for breast IMRT plans.^27^–^29^ Reduction in damage of the heart should also be a planning consideration as the use of some breast cancer chemotherapy agents such as anthracyclines can precipitate heart damage.^28^

## Conclusion

While the disadvantage of the IMRT SIB treatment technique is a slightly increased lung dose, this is contrary to findings in similar work. An IMRT SIB treatment technique when compared with CRT produces comparable plans for both the patient and the radiation therapy department.
